# Thromboelastography With Platelet Mapping is Not an Effective Measure of Platelet Inhibition in Patients With Spontaneous Intracerebral Hemorrhage on Antiplatelet Therapy

**DOI:** 10.7759/cureus.2515

**Published:** 2018-04-22

**Authors:** Helena Lam, Nakul Katyal, Catherine Parker, Prashant Natteru, Premkumar Nattanamai, Christopher R Newey, Chadd K Kraus

**Affiliations:** 1 University of Missouri School of Medicine, Columbia, USA; 2 Department of Neurology, University of Missouri, Columbia, USA; 3 Emergency Medicine, University of Missouri, Columbia, USA; 4 Department of Neurology, University of Mississippi Medical Center, Jackson, USA; 5 Neurology, Cleveland Clinic Ohio, Akron, USA; 6 Emergency Medicine, Geisinger Health System, Danville, USA

**Keywords:** intracerebral hemorrhage, platelet transfusion, platelet dysfunction, thromboelastography with platelet mapping

## Abstract

Thromboelastography with platelet mapping (TEG-PM) is a modality to measure platelet function, especially in patients taking antiplatelet medications. It consists of two components: arachidonic acid (AA), which is sensitive to aspirin, and adenosine diphosphate (ADP), which is sensitive to clopidogrel. In patients with spontaneous intracerebral hemorrhages (sICH), the clinical interpretation of platelet mapping is unclear. The objective of this study was to evaluate TEG-PM in patients with sICH on aspirin and/or clopidogrel who receive platelet transfusions. This study was an IRB-approved, retrospective case-control study over three years at an academic medical center. Adult patients with sICH were included if they had an admission computed tomography head (CTH) and platelet mapping followed by a repeat platelet mapping and CTH post platelet transfusion. A threshold of 50% inhibition was used as the benchmark for both ADP and AA inhibition. Around 248 subjects with sICH were identified, and 107 were excluded for incomplete documentation, leaving 141 for analysis. Of these, nine met our inclusion criteria. No statistical significance was found on the antithrombotic effects of aspirin or clopidogrel on TEG-PM (p=1.00 for both). Sensitivity and specificity of TEG-PM for clopidogrel was 100% and 42.9%, respectively, and 80% and 0%, respectively, for aspirin. Platelet transfusion did not significantly change AA or ADP inhibition (p=1.00). Hemorrhagic expansion on CTH was not associated with a decrease AA or ADP inhibition (p=1.00). TEG-PM is not an effective measure of platelet inhibition in sICH patients who were on antiplatelet medications and is not a reliable measurement following platelet transfusion.

## Introduction

Spontaneous intracerebral hemorrhage (sICH) is a devastating form of stroke that accounts for 6.5% to 19.6% of all strokes [1]. It has a 44% 30-day mortality rate with half of the deaths occurring in the first 48 hours. Greater hematoma volume and expansion are significant indicators of poorer prognosis [2]. sICH in the setting of anticoagulation, such as with warfarin, has predictable monitoring with an international normalized ratio (INR) [3]. However, treating sICH in the setting of antiplatelet therapy (APT) is less predictable. A systematic review in 2010 showed that patients who were taking APT had a 27% increased odds of death from sICH after one month compared to those not taking antithrombotic drugs [4]. Similarly, in a Finnish study, regular aspirin (ASA) use (median 250 mg) was associated with significant hematoma enlargement during the first week after sICH and two-fold increased mortality within the first three months [5]. A method to measure platelet function in sICH is desirable. Platelet transfusions are commonly used for APT reversal in the setting of active bleeding in sICH particularly if platelets <100,000 or if there is a need for emergent neurosurgical intervention [6]. However, platelet transfusion has not been found to reduce mortality [7]. A multi-center randomized trial (i.e., platelet transfusion versus standard care after acute stroke due to spontaneous cerebral hemorrhage associated with antiplatelet therapy (PATCH)) evaluated platelet transfusion within six hours from sICH for those on APT and found that there was an increased odds of death at three months (OR = 2.05, 95% CI:1.18-2.56; p=0.01) compared to the group who had received no platelet transfusion. The results of this study are significant and suggest that caution should be used prior to platelet transfusion for antithrombotic reversal [8]. There are multiple different platelet functions tests currently available, each with limitations [9].

Thromboelastograph® Platelet Mapping™ (TEG-PM) (Haemoscope Corporation, Niles, IL, USA) is a newer modality to measure platelet function. It is a global hemostasis test that is additionally able to monitor antiplatelet inhibition of arachidonic acid (AA) and adenosine diphosphate (ADP) [10]. Unfortunately, these markers are abnormal in brain injured patients regardless of antiplatelet use [11]. Further studies are needed to determine its reliability particularly in patients with sICH. Given the results of the PATCH trial along with our local use of TEG-PM, we evaluated the following: 1) the sensitivity and specificity of the platelet assay portion of TEG-PM in relation to antithrombotic usage in the brain-injured patient, and 2) the efficacy of the platelet assay portion of TEG-PM in response to platelet transfusion and ICH outcome.

## Materials and methods

**Figure 1:**
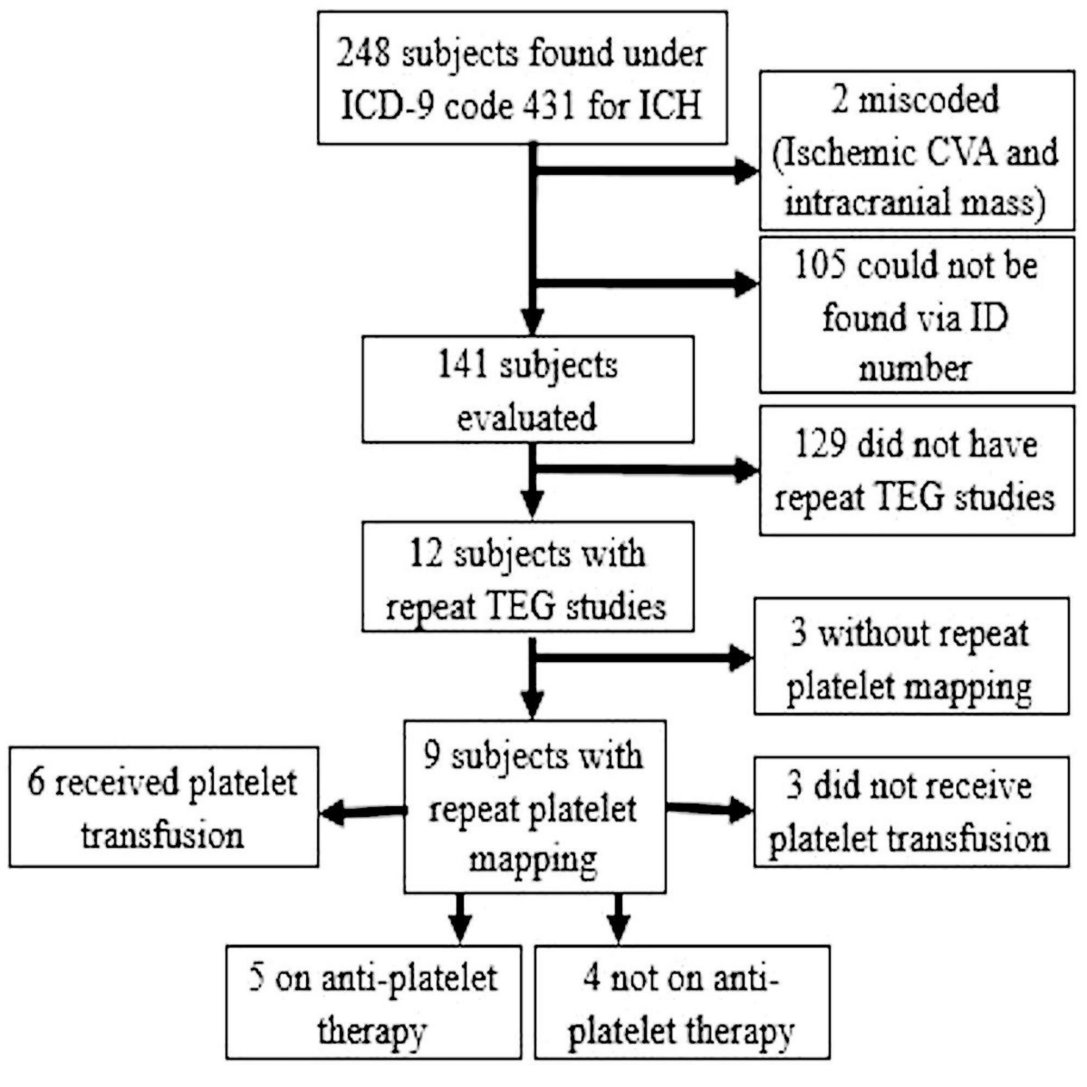
Study Design

In this retrospective case-control study, data from December 1, 2012 to October 1, 2015 was retrieved from an electronic medical records (Cerner, North Kansas City, MO) database from an academic, level trauma center using the ICD-9 code 431 for sICH. We included adult patients (>18 years of age) with sICH who had a computed tomography of the head (CTH) and an abnormal TEG-PM upon admission and prior to blood product correction along with a repeat TEG-PM and CTH. Patients were excluded if their charts were miscoded, had a mislabeled identification number, or had a lack of repeat TEG-PM. Figure 1 shows the breakdown of eligible subjects.

Baseline characteristics of the sample population included age, sex, weight (kg), comorbidities (hypertension (HTN), hyperlipidemia (HLD), diabetes mellitus (DM), congestive heart failure (CHF), and chronic kidney disease (CKD)), and antiplatelet therapy (none, aspirin, clopidogrel, or dual therapy). sICH related characteristics gathered include Glasgow Coma Score (GCS), National Institute of Health Stroke Score (NIHSS), ICH score, ICH volume from CTHs and subsequent CTHs, units of platelet transfusion, and other blood products received. Deep venous thrombosis (DVT) during hospital stay and the number of inpatient deaths were also collected.

Antithrombotic effect on TEG-PM

We analyzed ASA, clopidogrel (CLO), and dual antiplatelet therapy (DAPT) usage based on documented patient drug records with abnormal TEG-PM. Considering the local neurosurgical practice and findings of two different studies, a threshold of 50% inhibition was used as the benchmark for both ADP and AA in this portion of the study in order to determine the sensitivity and specificity of TEG-PM [12-13].

Platelet transfusion effect on TEG-PM

Decreases in ADP and AA inhibition from the initial lab and the repeat study were analyzed between the groups which had or had not received the platelet transfusion. These were then compared to determine the efficacy of TEG-PM for analyzing changes in platelet activity after reversal.

Outcome measurements

The frequency of hemorrhagic expansion obtained from reviewing the initial and repeat head CT in patients with abnormal TEG-PM results were analyzed. The ICH volume was calculated using the ABC/2 method, which is a quick method to obtain an accurate estimate of the volume [14]. The effect of platelet transfusion on ICH volume expansion was then determined.

Statistical analysis

Platelet inhibition is the extent of non-response of the ADP or thromboxane A2 receptor to ADP and AA, respectively. TEG measures the binding activity of activated platelets to a fibrin mesh and represents the maximal clot strength via measurements of maximal amplitude (MA). Platelet aggregation to agonist can, therefore, be calculated using the following formula: ((MAADP/AA – MAFibrin) / (MAThrombin - MAFibrin) x 100). Percentage platelet inhibition is therefore: 100% - % platelet aggregation. Using these formulas, the TEG-PM software calculates ADP % inhibition and AA % inhibition. AA is the agonist in the case of ASA therapy, whereas ADP is the agonist in CLO therapy [12]. Categorical data included ASA usage, CLO usage, DAPT, no APT, ADP inhibition of >50%, AA inhibition of >50%, initial and repeat ADP inhibition %, initial and repeat AA inhibition %, platelet transfusion, no platelet transfusion, and ICH expansion. Response to platelet transfusion was noted if there was at least a 20% difference from initial and follow-up AA and ADP values. The Fisher’s exact test was used to analyze our categorical data using a 2x2 contingency table. Odds ratios and confidence interval analyses were performed using a confidence interval (CI) of 95%. Continuous data were compared using t-test between the groups who had received and had not received platelet therapy.

All statistical analyses were performed using GraphPad Prism 7 (LaJolla, CA, USA). A p value of <0.05 was considered significant.

## Results

Two hundred and forty-eight patients were found to have sICH, but 107 were excluded due to incomplete documentation. Out of the 141 patient charts reviewed, nine of those patients met our inclusion criteria. Continuous variables between the group which had received platelet transfusion and the group which had not received platelet transfusion showed no statistically significant differences (p = 0.77; Table 1).

**Table 1:** Subject Characteristics Data are given as n (% total), median (range), or mean (+ SD). Yrs: years; Kg: kilogram; HTN: hypertension; HLD: hyperlipidemia; DM: diabetes mellitus; CVA: cerebrovascular accident; CAD: coronary artery disease; CKD: chronic kidney disease; ASA: aspirin; ICH: intracerebral hemorrhage; GCS: Glasgow coma score; NIHSS: National Institute of Health Stroke Scale; mL: milliliters; AA: arachidonic acid; ADP: adenosine diphosphate; DVT: deep venous thrombosis; NS: nonsignificant.

	Received platelet therapy	Did not receive platelet therapy	P value
Total (N=9)	6	3	1
Mean Age (years)	68.17(18.08)	57.33(17.62)	1
Male	5(55.6)	2(22.2)	1
Female	1(11.1)	1(11.1)	1
Mean Weight	78.27(9.28)	84.47(13.36)	1
Comorbidities			
HTN	5(83.3)	2(66.7)	1
HLD	2(33.3)	2(66.7)	1
DM	1(16.7)	1(33.3)	1
Prior CVA	1(16.7)	1(33.3)	1
CAD	2(33.3)	0	1
CKD	0	0	1
Antiplatelet therapy PRE-ICH			
ASA only	3(50)	1(33.3)	1
Clopidogrel only	0	0	1
ASA and clopidogrel	1(16.7)	0	1
None	2(33.3)	2(66.7)	1
ICH-related characteristics			
Median GCS score	9.67(3-15)	9.67(10-15)	1
Median NIHSS score	7.25(1-18)	12.67(2-20)	1
Median ICH score	2.17(1-4)	1(0-3)	1
Mean ICH initial volume (mL)	52.42(45.65)	8.94(6.63)	1
Mean initial AA inhibition (%)	78.5(14.9)	50.57(45.34)	1
Mean initial ADP inhibition(%)	50.22(36.2)	80.77(18.13)	1
Secondary Outcomes			
DVT complication during hospital stay	1(16.7)	0	1
Inpatient deaths	1(16.7)	0	1

Antithrombotic effect on TEG-PM: No statistical significance was found on the antithrombotic effects of ASA and CLO on platelet mapping (p=1.00 for both) (Table 2).

**Table 2:** Effect of Clopidogrel and Aspirin on ADP and AA Inhibition ADP: adenosine diphosphate; AA: arachidonic acid.

		Clopidogril Use (N=8)			Aspirin Use (N=8)		
		Yes	No	P value	Yes	No	P value
ADP inhibition >50%	Yes	1	4	1	NA	NA	NA
	No	0	3	1	NA	NA	NA
AA inhibition >50%	Yes	NA	NA	NA	4	3	1
	No	NA	NA	NA	1	0	1

Sensitivity and specificity of platelet mapping for CLO was 100% and 42.9%, respectively, and 80% and 0%, respectively, for ASA, using the benchmark of 50% AA and ADP inhibition.

Platelet transfusion effect on TEG-PM: Platelet transfusion did not significantly decrease AA (p=1.00, OR=0.75, CI 0.04-14.97) or ADP inhibition (p=1.00, OR=0.13, CI 0.01-3.23), per TEG-PM (Table 3).

**Table 3:** Platelet Transfusion or Hemorrhagic Expansion Associated with Reduction with ADP or AA ADP: adenosine diphosphate; AA: arachidonic acid.

		Platelet Transfusion (N=8)			Hemorrhagic Expansion (N=8)		
		Yes	No	P value	Yes	No	P value
Decrease in ADP	Yes	1	2	1	0	3	0.5
	No	4	1		2	4	
Decrease in AA	Yes	3	2	1	1	5	1
	No	2	1		1	2	

Outcome measurements: Hemorrhagic expansion on CTH was not associated with a decrease AA (p=1.00, OR=0.40, 95% CI 0.2-10.02) or ADP (p=0.50, OR=0.26, 95% CI 0.01-7.27) inhibition per TEGPM, respectively or with platelet transfusion (p=1.00, OR=0.40, 95% CI 0.2-10.02) as seen in Tables 3-4.

**Table 4:** Hemorrhagic Expansion or Thrombotic Complications Associated with Platelet Transfusion

		Hemorrhagic Expansion (N=9)			Death/Complications (N=9)		
		Yes	No	P value	Yes	No	P value
Platelet Transfusion	Yes	1	5	1	1	5	1
	No	1	2		0	3	

Secondary outcomes of DVT complication or inpatient deaths were not associated with platelet transfusion (p = 1.00; Table 4)

## Discussion

These results suggest that platelet mapping with thromboelastography is not a clinically useful modality to measure platelet inhibition in sICH patients who were on antithrombotics or to monitor changes in platelet function following platelet transfusion. A large contributing factor to its lack of efficacy appears to be due to its particularly poor specificity. These findings are similar to a recent TEG-PM study in acute trauma patients on APT in which a stricter benchmark of AA and ADP inhibition at 70% was used, and in a recent study following minor injury [15-16]. No precise level of platelet receptor inhibition has been established to determine clinical significance for platelet dysfunction or normal function due to wide ranges of inhibition seen in non-APT control groups. An AA and ADP inhibition range of 0% to 10% and 0% to 58%, respectively, have been found in healthy blood donors (n=43) [13]. Also, a mean AA and ADP inhibition of 17.5% and 47.8% respectively have been found in non-APT pre-operative patients (n=59) [12]. These former findings agree with the lack of specificity and limitation of the TEG-PM system to be used independently as a platelet function test in these scenarios. The level of inhibition seen in subjects not on APT suggests that there may be other factors altering platelet function or interacting with platelet receptors. Some factors include altered physiology in trauma, alcohol consumption, certain foods, such as garlic, and temperature changes [11-12,17]. Increased ADP inhibition (64.5% vs 15.5%; p<0.01) and AA inhibition (25.6% vs 2.2%; p<0.01) in isolated head injuries unrelated to APT were found compared to an uninjured group [11]. The average ADP inhibition seen with traumatic brain injury (TBI) has been found to be 86.5% to 93.1% with a GCS<8 and 40.4% to 49.8% with a GCS>8 compared to 15.5% of the controls [11,18]. This increased level of ADP inhibition with TBI appears to correlate with greater quantities of dense granules seen via flow cytometric assays [19]. In a study of 459 patients with minor injury, there was no correlation of TEG-PM with injury severity score (ISS), length of stay (LOS) or mortality despite the finding of normal clot strength with abnormal AA and ADP inhibition of 30% and 58%, respectively [16]. Despite these recent findings on the effects of trauma on platelet receptor inhibition, it is still uncertain as to whether these findings apply to the non-traumatic spontaneous cerebral injuries. TEG-PM was not compared to other current platelet function tests in this study. VerifyNow-ASA and PFA-100 have been previously evaluated. Forty-two percent of patients assessed with the VerifyNow assay and 52% assessed with the PFA-100 assay were found to have abnormal platelet function without any history of antiplatelet use. Overall, the agreement between the two assays is poor (k=0.26, p=0.07) [9]. Past studies have shown TEG-PM to be comparable to optical platelet aggregometry and superior to PFA-100 in the assessment of ADP receptor function [12]. Further comparison studies would benefit the evaluation of TEG-PM reliability in such context. Platelet transfusion, such as what we showed in our study, was not found to be associated with significant changes in sICH volume expansion, suggesting a lack of outcome benefit from receiving a platelet transfusion. This finding contrasts with that by Naidech et al. who found that platelet transfusion improved platelet activity (as measured by the VerifyNow-ASA assay) resulting in a smaller final hemorrhage size [20]. Recently, the PATCH trial, which was a multicenter trial, evaluated platelet transfusion to standard care in patients with ICH who were on APT. One hundred ninety patients were enrolled over 80 months. The primary findings of odds of death or dependence at three months were higher in the platelet-transfused group (p=0.01, OR 2.05, 95% CI 1.18-3.56) [8]. In a separate study in elderly patients, the transfusion of platelets did not reduce mortality [7]. Given these findings, platelet transfusion cannot be recommended for patients with ICH who were on APT.

This study has expected limitations associated with a retrospective chart review. The determination of APT use was dependent on documented medication records received by the hospital. Many charts were excluded due to errors in coding and lack of repeat TEG-PM studies. Other possible confounders included transfer admissions and direct admissions to the center’s emergency department. Approximately one-third of the included subjects were transferred from other hospitals. Additionally, our sample size was small, and future studies with larger populations could help to determine dose-dependent changes of AA and ADP inhibition and other influential factors to their levels on TEG-PM. The lack of significance with the use of TEG-PM following platelet transfusion is even more relevant given that patients who received platelet transfusion had lower NIHSS.

## Conclusions

The use of platelet mapping with thromboelastography appears to be limited by its nonspecific findings of platelet receptor inhibition in determining anti-platelet therapy, determining postplatelet transfusion changes, and predicting hemorrhagic expansion outcomes in patients with sICH. Platelet transfusion cannot be recommended for the sole purpose of APT reversal in ICH patients based on TEG-PM.
